# Cone and rod cells have different target preferences in vitro as revealed by optical tweezers

**Published:** 2008-04-21

**Authors:** Robert J. Clarke, Kormákur Högnason, Michael Brimacombe, Ellen Townes-Anderson

**Affiliations:** 1 Department of Neurology and Neuroscience, New Jersey Medical School, University of Medicine and Dentistry of New Jersey, Newark, NJ; 2 Department of Pharmacology and Physiology, New Jersey Medical School, University of Medicine and Dentistry of New Jersey, Newark, NJ;; 3 Department of Preventive Medicine and Community Health, New Jersey Medical School, University of Medicine and Dentistry of New Jersey, Newark, NJ

## Abstract

**Purpose:**

When neural circuits are damaged in adulthood, regenerating and sprouting processes must distinguish appropriate targets to recreate the normal circuitry. We tested the ability of adult nerve cells to target specific cells in culture using the retina as a model system.

**Methods:**

Under sterile culture conditions, retinal cells, isolated from tiger salamander retina, were micromanipulated with optical tweezers to create pairs of first-order photoreceptor cells with second- or third-order retinal neurons. The development of cell contact and presynaptic varicosities, the direction and amount of neuritic growth, and nerve cell polarity were assessed after seven days in vitro. Cultures were labeled for rod opsin to distinguish rod from cone cells and for the alpha subunit of the trimeric G protein Go (Goα) to identify cone-dominated and mixed rod-cone ON bipolar cells.

**Results:**

Quantitative analysis of growth demonstrated that target preferences were cell-specific: Cone cells preferred second-order bipolar cells, whereas rod cells grew toward third-order neurons, which include amacrine and ganglion cells. In addition, when rod cells grew toward bipolar cells, they chose an abnormally high number of Goα-positive bipolar cells. These growth patterns were not affected by tweezers manipulation or the amount of growth. Cell orientation of the photoreceptor also did not affect preferences: Cells oriented away from dendritic processes could reorient their axonal pole toward the target cell.

**Conclusions:**

Cone cells preferred normal partners, and rod cells preferred novel partners. These intrinsic preferences indicate that adult nerve cells can have differing capacities for targeting even if they come from the same cell class. Further, these differences may help explain the patterns of photoreceptor sprouting seen in retinal degeneration in which rod, but not cone, cells invade the inner retinal layers where third-order neurons are located.

## Introduction

Subsequent to neuronal determination, the differentiating nerve cell produces an axon that grows with relative accuracy to its designated postsynaptic cell. Target selection, which must occur before synaptogenesis, occurs in several steps, including defasiculation (for projection neurons), branching in the target region, finding the correct topographic location, terminating in the appropriate layer, and connecting with the appropriate cells within that layer [1]. These carefully orchestrated activities result in cell-specific patterns of connectivity.

In regeneration of the central nervous system (CNS), additional activities by growing processes must occur. Axons need to overcome mechanisms of inhibition, which are established after the brain and spinal cord are developed [2,3]. Once axonal outgrowth is achieved, there must be target selection by the new processes for functional recovery. Both regrowth of adult axons and correct targeting depend upon environmental and intrinsic factors. Not only must external cues be present, but the cell must be able to respond to these cues. Selective axonal targeting by fetal mammalian cells transplanted into injured adult brain suggests that necessary environmental cues are present [4]. However, even in the well known retinotectal pathway of the goldfish, regenerating axons make many errors in their initial target area selection [5]. In the lizard, retinal ganglion cells grow to the tectum but are unable to find correct topographic locations to terminate [6]. Thus, in repair of injury to the CNS, targeting may not proceed smoothly even when inhibition of growth has been overcome.

**Figure 1:**
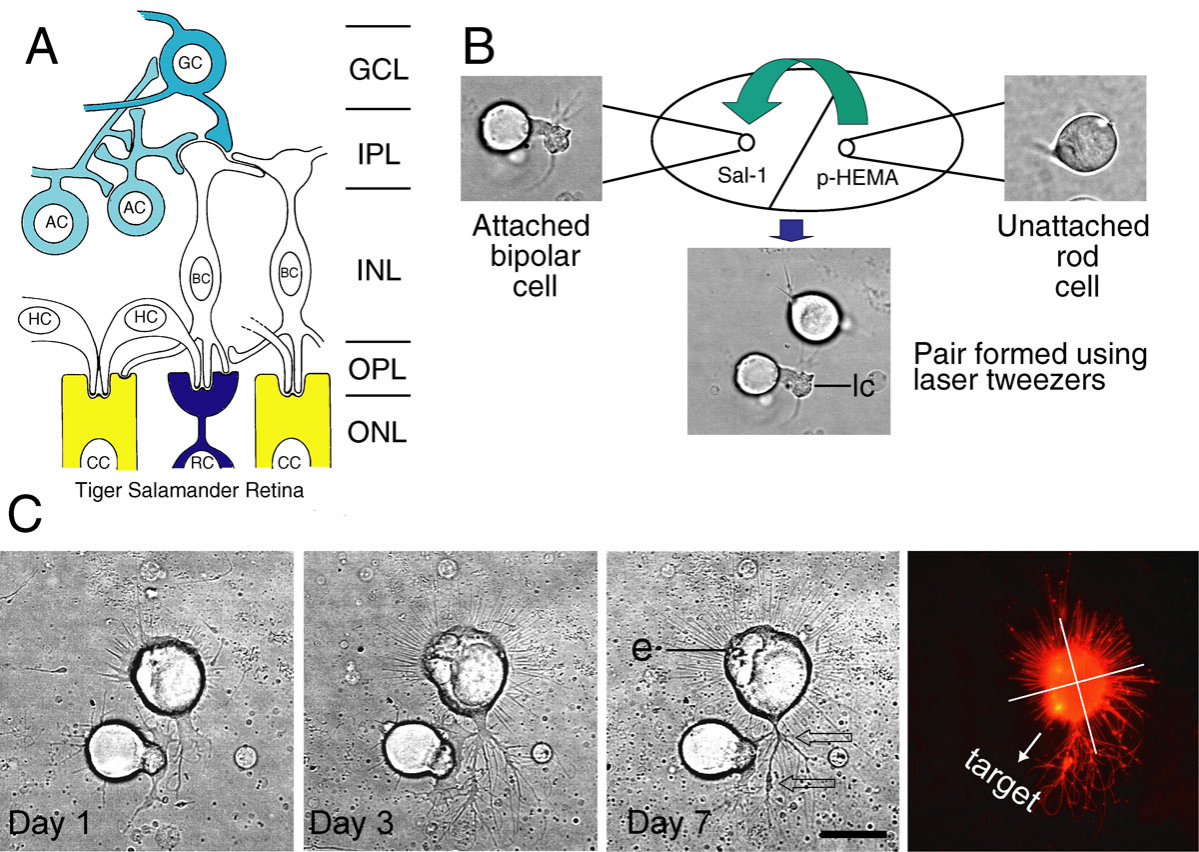
Movement and culture of cells grouped as pairs with optical tweezers. **A:** Schematic organization of the salamander retina based on Lasansky [9,10] and Wong-Riley [11]. Rod (RC) and cone cells (CC) connect with second-order neurons, horizontal (HC) and bipolar cells (BC), which in turn connect with third-order neurons, amacrine (AC) and ganglion cells (GC). Normal interactions for photoreceptors, therefore, are exclusively with second-order neurons. **B:** Creating cell pairs with laser tweezers. A rod cell on the poly-HEMA side of the culture dish (upper right, see Methods for more details) is selected, trapped in the laser beam and transported to the Sal-1 side where it is placed less than 10 µm away from a bipolar cell (upper left) to form a pair (lower middle). The bipolar cell was identified by the presence of a thick Landolt club (lc). The photoreceptor was identified by the presence of an ellipsoid best seen in part **C** (e). **C:** Analysis of growth. Growth was followed for one week in culture. Photoreceptor identification, initially made by the presence of an ellipsoid (e) and cell shape, was confirmed by immunopositive staining for rod opsin (far right). This rod photoreceptor is attracted to the bipolar cell. At day 1, a thick lamellar process forms from the photoreceptor cell, growing toward neurites emanating from the bipolar Landolt club. By day 7, long neuritic processes with varicosities (arrows) were present; some processes were contacting the dendrites of the target cell. Quantitative analysis of growth in the quadrants toward and away from the target (far right) verified that, in addition to multiple contacts, there were more varicosities toward the target. Scale bar equals 20 µm.

Many areas of the CNS consist of multiple cell layers, such as the cortical and cerebellar layers, layers of the spinal dorsal horn and thalamic nuclei, and retinal layers. Progress is being made in understanding how neurons target their axons to specific layers in these structures during development (for a review, see [7]). However, little is known about targeting to specific cells and therefore layer-specific recreation of connectivity in the adult after injury. Some of the lack of information regarding injury repair in the adult CNS is due to the absence of model systems where targeting can be examined at the cellular level. We used a culture system of adult amphibian retinal cells that can be maintained in defined medium and in which functional synapses form [8]. The aim of the present study was to examine the ability of adult sensory neurons, the cone and rod photoreceptors, to regenerate appropriate circuitry by assessing the targeting of photoreceptor axon outgrowth. The intact retina consists of three cellular layers separated by two synaptic layers. Cone and rod photoreceptors synapse with second-order horizontal and bipolar cells in the outer synaptic layer. The bipolar cells, in turn, interact with the third-order (amacrine and ganglion) cells in the inner synaptic layer (Figure 1A, based on Lasansky [9,10] and Wong-Riley [11]). This structure is consistent across all vertebrate species. Thus, correct targeting of adult photoreceptors would result in interactions with second-order neurons, exclusively.

The issue of targeting in the adult retina takes on additional significance due to reports of neuritic sprouting by adult photoreceptors. This growth has been observed in a variety of human retinal degenerations including retinitis pigmentosa (RP), age-related macular degeneration, and retinal detachment (reviewed in [12,13]). In particular, rod, but not cone, cells grow neurites with presynaptic varicosities filled with synaptic vesicles into the inner retina, where the amacrine and ganglion cells are located. In an animal model of one form of RP, cone cell neuritic growth has been observed but much of this sprouting remained in the outer retina [14]. The cause of this sprouting and the functional consequences to the diseased retina are unknown.

In a previous study [15], which examined randomly plated retinal neurons after two weeks in culture, we discovered a statistically significant preference for novel third-order neurons as synaptic contacts of photoreceptor cells. This study suggested that in the retina, at the level of cell recognition, correct targeting by photoreceptors did not occur. However, random platings of cells presented several technical problems. To insure the formation of cell pairs and groups, we plated the cultures at relatively high density, allowing multiple cells to interact with an individual photoreceptor. The cellular influences on these preferences, therefore, were probably multivariate, making it difficult to know which cells or secreted cell products influenced targeting and contact formation. Additionally, it was not possible to identify all second- and third-order neurons. This led to significantly fewer groups with identifiable bipolar cells. Finally, cone and rod photoreceptors were not analyzed separately, in part because they were difficult to distinguish morphologically after two weeks in culture.

We have overcome these issues with the use of optical tweezers. Optical tweezers work by trapping a cell in a beam of infrared laser light. We have previously shown that retinal cells can be manipulated by laser light without toxicity [16]. In the present study, tweezers were used to create pairs of cells, where both the photoreceptor cell type and the class of the potential target cell, either a second-order bipolar cell or a third-order multipolar cell, were identified from the outset of culture. Micromanipulation by optical tweezers allowed the maintenance of sterility: because the laser beam goes through transparent surfaces of the culture dish, cell selection and placement could be done in an enclosed, sterile environment. Cultures could be low density because pairings depended on micromanipulation, not chance association. Additionally, distances between cells could be standardized because of the micron-level control of tweezers micromanipulation.

By examining cultures at seven days, we were able to focus on the question of targeting by regenerative neuritic growth rather than synaptogenesis. This study confirms and expands our prior work: cone cells are attracted to their normal targets, but rod cells are attracted by novel targets, thereby suggesting that the ability to appropriately target cells after injury is cell-type dependent. In addition, the methodology describes a unique culture system, where selected cell-cell interactions can be reliably tested, bringing to fruition the initial promise of optical tweezers in nerve cell manipulation [16].

## Methods

### Preparation of culture dishes for optical tweezers

Trapping forces of optical tweezers are generated from the momentum of light [17,18]. Although these forces easily trap cells in suspension, they are not able to move cells that adhere to a surface. In initial experiments to manipulate retinal neurons with optical tweezers [16], we used a thin layer of Sylgard (described in the following paragraph) to reduce cell adhesion to the culture dish. For the current studies, we developed a new technique to reduce cell adhesion using poly-2-hydroxyethylmethacrylate (poly-HEMA), a nontoxic compound with cell repellent properties previously employed for adhesion studies on endothelial cells [19].

An acid-cleaned #1 glass coverglass (VWR Scientific Inc., Media, PA) was prepared so that one half was coated first with 20 mg/ml poly-HEMA (Sigma Chemical Co., St Louis, MO) in 95% ethanol. Allowing a few drops of poly-HEMA solution to flow down the surface of the coverglass held at a steep incline ensured a thin, even coating. After drying in air in a dust-free environment, the coverglass was glued with Sylgard 184 (Dow Corning Co., Midland, MI) over a 1 cm hole that had been drilled in the bottom of a 35 mm culture dish. A line indicating the edge of the poly-HEMA coating and two fiducial points to act as reference points for locating cell position were scratched on the bottom of the dish. The dishes were sterilized with ultraviolet light overnight. The other half of the coverglass was made adhesive to salamander retinal neurons by coating with 0.1 mg/ml sterile goat anti-mouse IgG antibody (Boehringer Mannheim Corporation, Indianapolis, IN) and then, after rinsing with sterile Ringer’s, coating with Sal-1 supernatant containing mouse anti-salamander antibody raised against retinal cell membranes (generously provided by Dr. Peter MacLeish, Morehouse School of Medicine, Atlanta, GA; see MacLeish et al. [20]). Dishes were incubated at 10 °C overnight. They were then rinsed with Ringer’s to remove Sal-1 before introduction of 2 ml serum-free salamander medium, which contained 108 mM NaCl, 2.5 mM KCl, 2 mM HEPES, 1 mM NaHCO_3_, 0.5 mM NaH_2_PO_4,_ 1 mM sodium pyruvate, 0.5 mM MgCl_2_, 24 mM glucose, 1.8 mM CaCl_2_, 7% medium 199, 1 mM minimum essential medium (MEM) vitamin mix, 0.1 mM MEM essential amino acids, 0.1 mM MEM nonessential amino acids, 2 mM glutamine, 2 µg/ml bovine insulin, 1 µg/ml transferrin, 5 mM taurine, 0.8 µg/ml thyroxine, 10 µg/ml gentamycin, and 1.0 mg/ml BSA [8,21].

**Figure 2:**
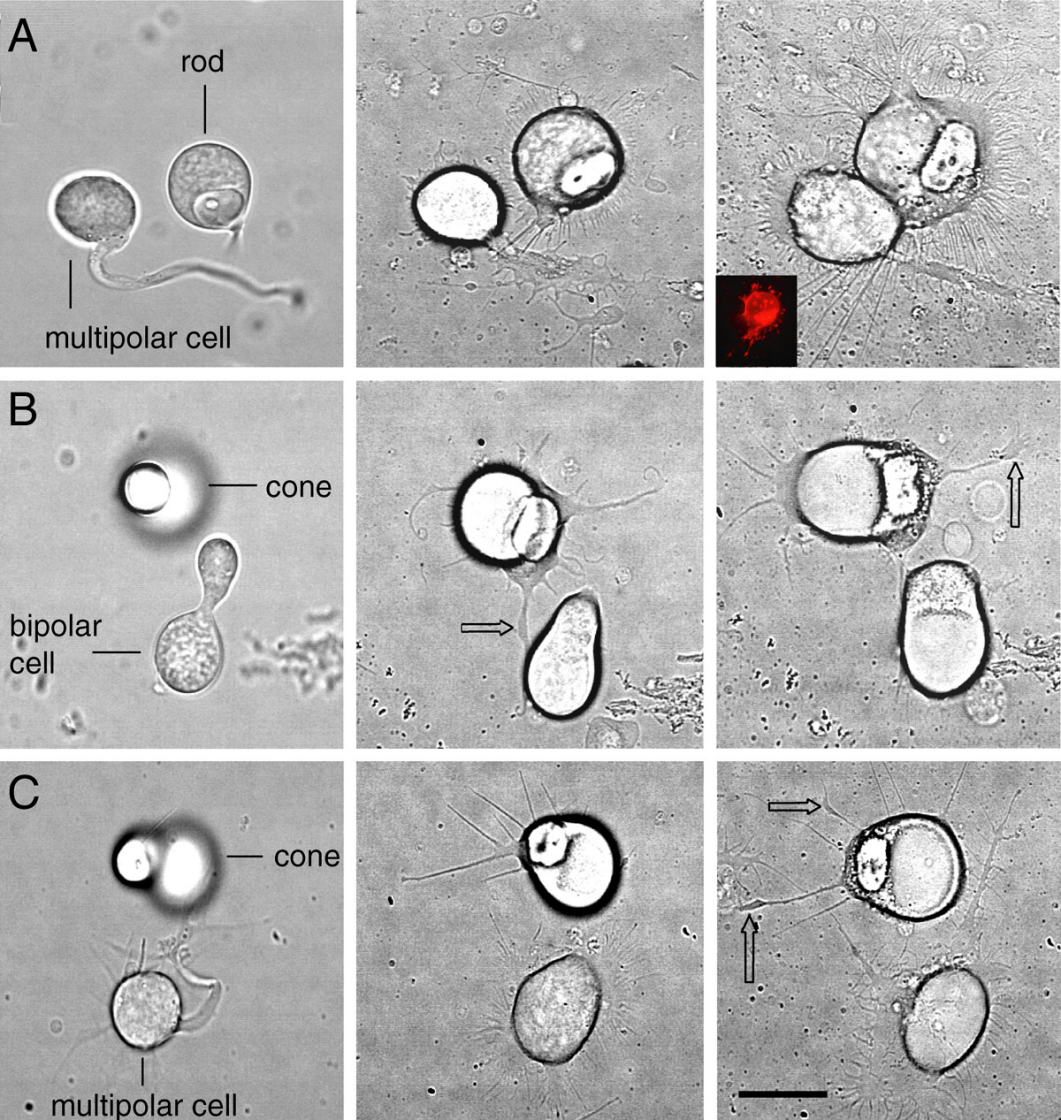
Normal and novel interactions after laser tweezers micromanipulation. The left column shows cells immediately after tweezers placement; the middle column, 3 days in vitro; and the right column, 7 days in vitro. **A:** An example of attraction between a rod cell and a multipolar cell. At 3 days, long filopodial processes emanating from the rod cell contacted processes of the multipolar cell. The cells grew toward each other over 7 days in culture creating a broad intercellular contact. Inset: The photoreceptor identification as a rod cell was confirmed by immunolabeling with anti-rod opsin. **B:** An example of attraction between a cone cell and a bipolar cell. At 3 days, a neurite bearing a varicosity (arrow) contacted the bipolar cell. A second varicosity developed by day 7. For cone cells, quantitative analysis compared the two halves of the cell for growth since cone cells grew fewer processes than rod cells. Greater numbers of neurites and varicosities (arrows) on the side facing the bipolar are indicative of attraction between the cells. **C:** An example of repulsion between a cone cell and a multipolar cell. The larger numbers of neurites and varicosities (arrows) on the opposite side of the cone with respect to its paired multipolar cell indicate repulsion. Scale bar equals 20 µm.

### Preparation of cell cultures

Retinal cell cultures were obtained from light-adapted, adult, aquatic-phase tiger salamanders (*Ambystoma tigrinum*), measuring 17–22 cm in length (Charles Sullivan Inc., Nashville, TN). All protocols were approved by the Institutional Animal Care and Use Committee at the University of Medicine and Dentistry of New Jersey and were in strict accordance with the guidelines from the National Institutes of Health. The animals were maintained at 5 °C on a 12 h:12 h light-dark cycle for at least one week before experimentation. Cultures of retinal cells were prepared according to procedures described by Nachman-Clewner and Townes-Anderson [22]. Briefly, the animals were decapitated and pithed, and the retinas removed in room light. The retinas were subjected to enzymatic digestion with 14 U/ml papain (Worthington, Freehold, NJ) in salamander Ringer's solution (85 mM NaCl, 1.5 mM KCl, 25 mM NaHCO_3_, 0.5 mM CaCl_2_, 0.5 mM NaH_2_PO_4_, 24 mM glucose, 0.03 mM phenol red, 1.0 mM sodium pyruvate) containing 2.7 mM DL-cysteine for 45 min at room temperature (20–22 °C). Retinas were rinsed with Ringer’s solution and then gently triturated with a 3 mm bore pipette to yield a cell suspension containing a mixture of photoreceptors, second- and third-order neurons, as well as Müller cells.

**Figure 3:**
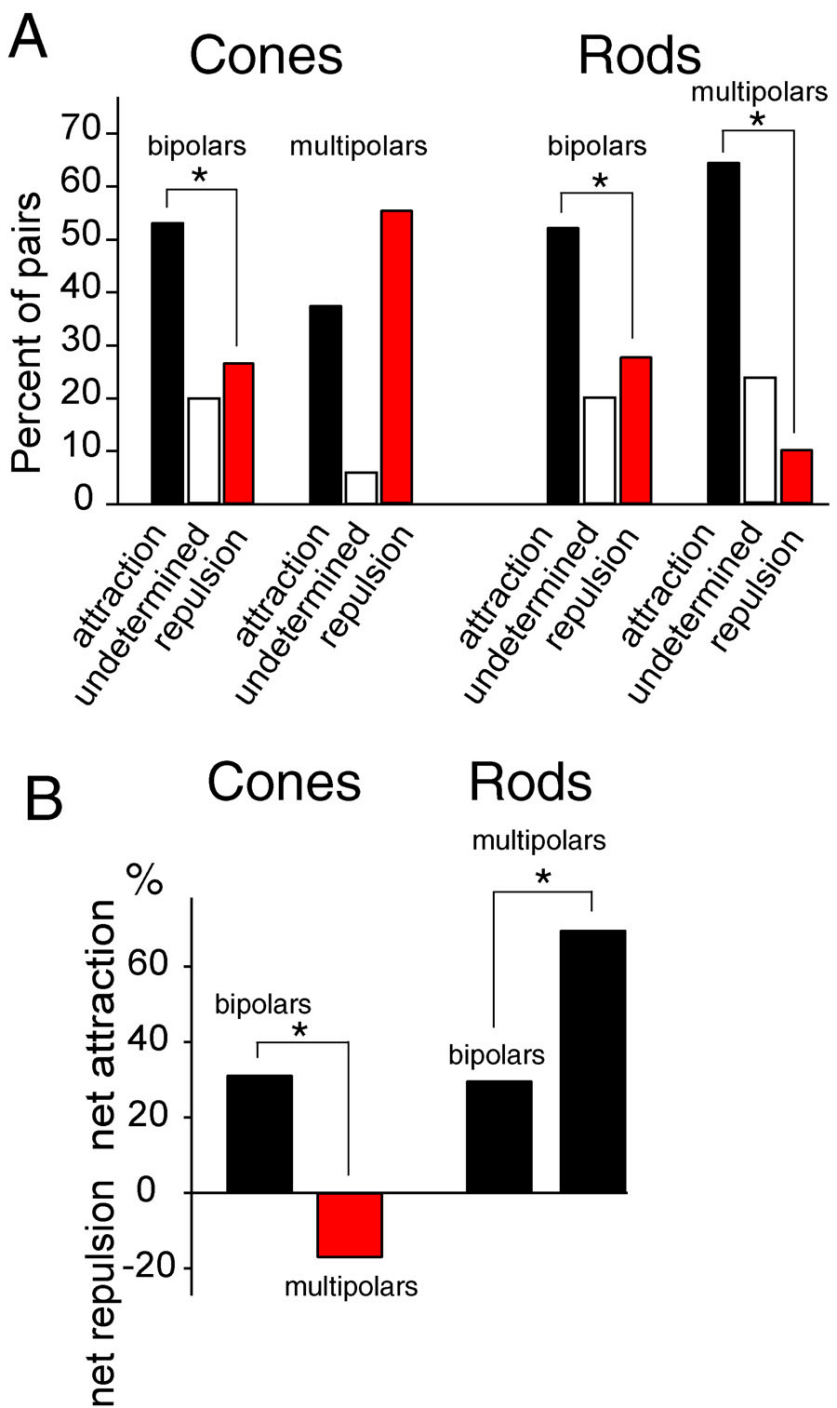
Analysis of attraction and repulsion between photoreceptors and their target cells. **A:** Cell pairs were classed as either showing attraction, repulsion, or undetermined, which means showing neither attraction nor repulsion. There were 89 pairs with cone cells and 114 pairs with rod cells. Cone cells were significantly more attracted than repulsed by bipolar cells. Rod cells were significantly more attracted than repulsed by bipolar cells and, when paired with multipolar cells, were also significantly more attracted than repulsed by them. Significance (asterisk denotes p<0.05) was tested with the exact binomial test. **B:** The net percent of attraction or repulsion was computed by subtracting the percent of repulsed pairs from the percent of attracted pairs. Although rods were attracted to both multipolar and bipolar cells, they were significantly more attracted to multipolar cells than bipolar cells. Cones, on the other hand, were more attracted to bipolar than multipolar cells. Significance (asterisk denotes p<0.05) was determined with the Pearson χ^2^ test.

### Laser tweezers manipulation of retinal cells

The optical tweezers-microtool or laser tweezers used for manipulation (Cell Robotics Inc., Albuquerque, NM) consisted of a 1 W, continuous wave diode laser of 980 nm wavelength mounted on an Axiovert 100 inverted light microscope (Carl Zeiss Inc., Thornwood, NY). Laser light was transmitted to the cells via an objective lens and thus was focused at the same focal plane as the microscope. A high numerical aperture (N.A. 1.3) 40x oil immersion plan neofluor objective (Carl Zeiss Inc.) was used with bright-field optics for optical trapping. Computer software (Cell Robotics Inc.) controlled laser power and movement of the motorized stage, stored microscope stage coordinates, and created macros for cell movement. A culture dish was placed on the microscope stage and cells were plated onto both halves of the prepared coverglass and allowed to settle for approximately 20 min, which is long enough for cells on the adherent side of the dish to attach to the antibody substrate.

Freshly isolated cells were identified predominately by cell shape [15]. Third-order neurons, the multipolar amacrine and ganglion cells, were identified by the presence of one or more processes emanating from the cell body. Amacrine and ganglion cells were not distinguished from each other in this study; these neurons were classed as multipolar cells. Bipolar cells in salamander retina have a large primary dendrite known as a Landolt club from which secondary dendrites emerge. This feature is characteristic of all bipolar neurons. Horizontal cells, although identifiable, were not used in this study because of their low abundance. Photoreceptors were identified by the presence of the ellipsoid, an accumulation of mitochondria, in the inner segment. Cone and rod cells were distinguished from each other by overall shape, hourglass versus rounded respectively, and for the rod cells, when retained, the presence of an axonal fiber with synaptic pedicle(s). For cone cells, there is no axon fiber and all cone cells, therefore, were assumed to have their presynaptic terminal, which lies at the nuclear pole.

Cell pairs were created by selecting an isolated second-order bipolar, or third-order amacrine or ganglion cell on the Sal-1 adherent side of the dish (Figure 1B). X and y stage coordinates of a position approximately 10 µm from the primary dendrites or Landolt club of the selected cell were marked and saved in the computer. A photoreceptor was then found on the nonadherent poly-HEMA side of the dish and optically trapped in the laser tweezers. Only photoreceptors without outer segments were selected for study. Although trapping could be achieved over a broad range of power levels, for our micromanipulations we routinely used the laser at 10%–20%, a setting low enough to avoid trapping debris but sufficient to transport the cell through the medium. While holding the photoreceptor cell, we lowered the stage so that the cell was well above the surface of the culture dish and any attached neurons. The stage was then moved under computer control to bring the cell to the previously saved x and y coordinates. Stage movement was set at 8–20 µm/s. Finally, the stage was raised to bring the trapped photoreceptor to the surface of the dish. The cell was placed within 2–10 µm of the selected cell’s processes where it was allowed to adhere to the Sal-1 substrate. Digitized images of both cells in the pair before and after pair formation with the laser tweezers were obtained with a black and white CCD camera (Sony Corporation, Tokyo, Japan) mounted on the microscope. The cell pairs were maintained in a humidified chamber at 10 °C in the dark for seven days. Each newly formed pair was daily monitored for growth. On the seventh day, cultures were fixed for immunocytochemistry for further identification.

### Immunocytochemistry

Rod and cone cell identification was confirmed by the presence and absence, respectively, of rod opsin immunostaining using the monoclonal antibody 4D2 [23] (a gift of Dr. Robert Molday, University of British Columbia, Vancouver, Canada). This antibody recognizes the opsin in red (M) rod cells, which comprise 98% of all salamander rod cells [24]. A mouse monoclonal antibody against the alpha subunit of the trimeric G protein Go (Goα; MAB #3073, Chemicon International, Temecula, CA) was used to stain ON bipolar cells [25]. Both antibodies have been previously characterized in salamander retina [21,26]. Cells were fixed with 4% paraformaldehyde in 0.125 M phosphate buffer, pH 7.4, for 24 h at 4 °C. Procedures for immunocytochemistry have been previously reported [21]. Briefly, cells were washed with PBS (450 mM NaCl, 20 mM sodium phosphate buffer, pH 7.4), then incubated in goat serum dilution buffer (GSDB; 16% normal goat serum, 450 mM NaCl, 0.1% Triton X-100, 20 mM phosphate buffer, pH 7.4) to block nonspecific binding and permeabilize the plasma membrane. The cells were then incubated with the primary antibody (4D2 1:25, Goα 1:1000 dissolved in GSDB) and maintained at 4 °C overnight. For negative controls, no primary antibodies were added to the GSDB at this step. Cells were rinsed with wash buffer (450 mM NaCl, 0.3% Triton X-100, and 20 mM phosphate buffer) followed by a rinse with PBS. Then the cells were incubated with Triton-free GSDB for 1 h at room temperature followed by incubation with Alexa 594-conjugated goat anti-mouse IgG (1:35, Molecular Probes, Eugene, OR) secondary antibody in Triton-free GSDB for 60 min at room temperature in the dark. Cells were washed with PBS followed by a final rinse with 5 mM phosphate buffer, pH 7.4, and mounted in antifade medium consisting of 90% glycerol, 10% PBS, and 2.5% (w/v) 1,4-diazobicyclo-2,2,2-octane.

**Figure 4:**
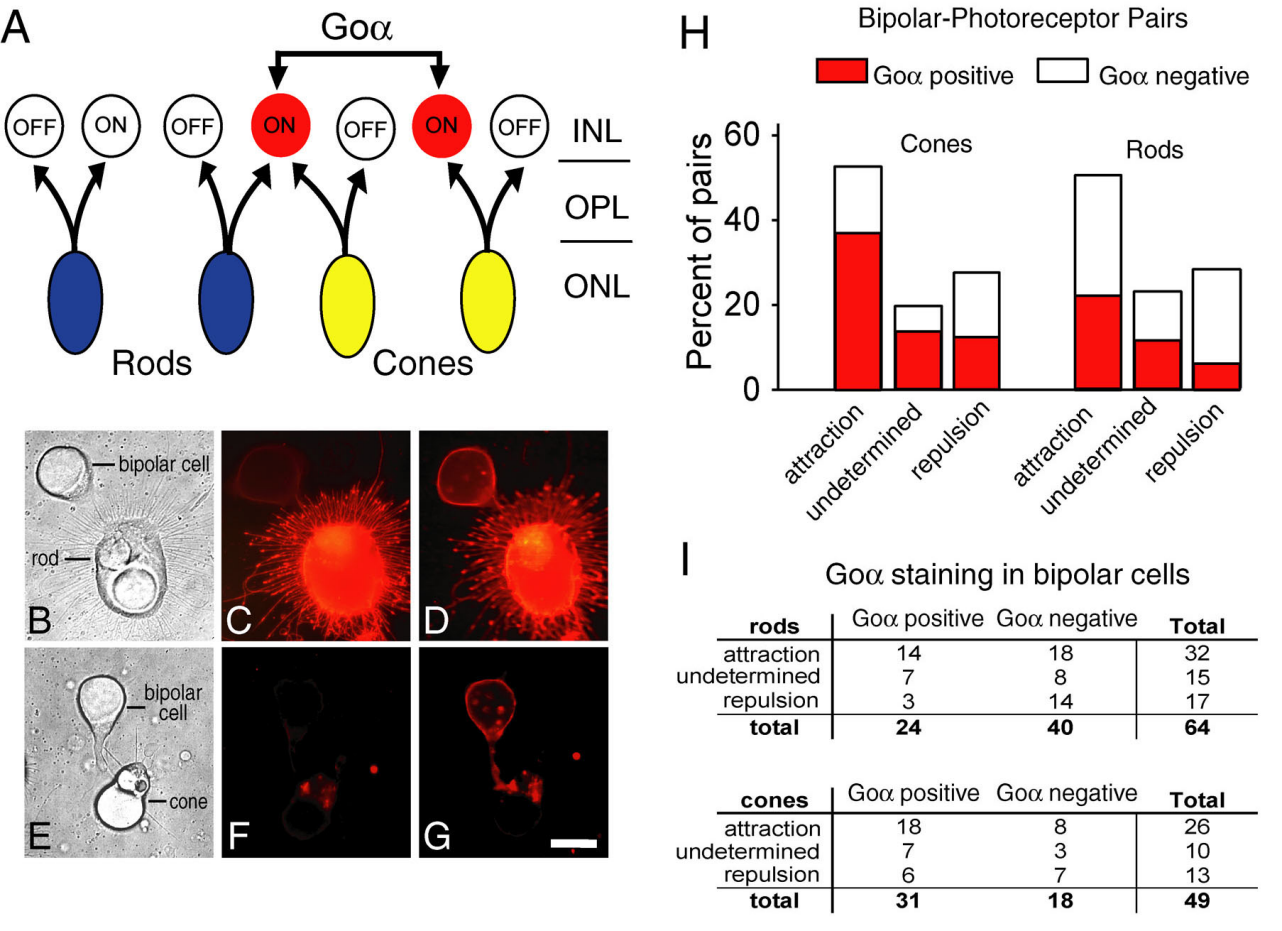
Photoreceptors paired with bipolar cells, analyzed for ON and OFF subtype. **A:** Schematic diagram of the types of bipolar cells and their inputs in salamander retina (based on [26,30,31]). ON bipolar cells with cone input stain positive for Goα (red). All cone cells contact Goα-positive bipolar cells. In contrast, for rod cells, only 30% contact Goα-positive cells. Thus, some of the ON bipolars contacted by rod cells are not Goα-positive. Both cone and rod cells also contact OFF bipolar cells. **B-G:** Detection of Goα-positive bipolar cells: **B:** A pair consisting of a rod and a bipolar cell was classified as undetermined after 7 days in culture, i.e., the photoreceptor did not show more growth either toward or away from the target bipolar cell. **C:** The rod cell stained immunopositive for rod opsin confirming its identification as a rod cell. **D:** The same pair, after staining for Goα, showed that the bipolar cell stained immunopositive for Goα. Goα immunolabel is present along the cytoplasmic surface of the plasma membrane in all parts of the cell as has been described for salamander bipolar cells [26]. **E:** A pair consisting of a cone cell and a bipolar cell was classified as attractive after 7 days in culture. The attraction was evident due to more neuritic growth toward than away from the target. **F:** After staining for rod opsin, the cone was immunonegative confirming its identification. **G:** After Goα staining, the bipolar cell was immunopositive whereas the cone cell retained only background staining. Scale bar equals 20 µm. **H:** Goα-positive and -negative bipolar cells were distinguished in pairs made with cones (n=49) and rods (n=64) for the attracted, repulsed and undetermined categories (see **I** for the actual numbers in each group). Cone and rod cells were both attracted to and repulsed by Goα-positive bipolar cells. However, for both cone and rod cells, Goα-positive bipolar cells were significantly more attractive than repulsive (p<0.03 and p<0.02, respectively). **I:** In vivo about 30% of rod cells contact Goα-positive bipolar cells [26]. In cultured pairs, the number of rod cells that were attracted to Goα-positive bipolar cells was significantly higher than expected: about 82% (14 of 17 cells) of Goα-positive cells were attractive to rod cells (p=0.001, tested with the exact binomial test). Pairs classified as undetermined were not included in the analysis. The proportion of Goα-positive bipolar cells of the total bipolar cell population is 41% in vivo. The proportion of Goα-positive to Goα-negative cells presented to rod cells was similar, 38% (24/64) but for cone cells it was 63% (31/49). See text for discussion.

### Analysis of photoreceptor cell growth

Analysis was performed on the digitized images of cone and rod cells taken using conventional phase-contrast and fluorescence microscopy, respectively. Because of the larger numbers and finer processes of rods, it proved easier to carry out measurements on the immunostained cells. Any outgrowth extending >5 µm from the soma or from lamellipodial-like processes extending from the soma was considered to be a primary process. Formation of a varicosity along a primary process demonstrated differentiation into a neuritic process. These varicosities are known to be filled with synaptic vesicles capable of recycling in response to depolarization [21]. For quantification, a varicosity was defined as a swelling along a neuritic process with a diameter >1µm [21]. Distance between cells after plating and again after seven days in vitro was examined along the axis connecting the nuclei. Measurements were made double blind using Image-Pro Plus v4.1 (Media Cybernetics, Silver Spring, MD).

To determine attraction and repulsion, we drew a straight line, connecting the centers of both cells in a pair. Cell center was determined by averaging the x and y axes. Cones were then divided into halves by drawing a single line through the cell’s center that intersected the first line at right angles. Because rod cells have many more processes [27], they were divided into equal-sized quadrants by superimposing a cross consisting of two lines each subtending 45 degrees with respect to the line joining the cells’ centers and intersecting at the photoreceptor cell center (see Figure 1C). Measurements of cell growth were made within the half (cones) or quadrant (rods) facing toward the target cell and away from the target cell and then compared. Whether a photoreceptor was attracted or repelled by the target cell was determined by (a) examining for cell contacts, (b) calculating the difference between the number of presynaptic varicosities formed by the photoreceptor in the direction toward and away from the target neuron, (c) calculating the difference between the number of primary neurites and the total neurite length toward or away from the target, and (d) examining for reduction or expansion of the distance between the photoreceptor and paired second- or third-order cell bodies. The formation of contact and the location of varicosities were of primary importance in determining attraction; thus, growth that resulted in broad contact between the photoreceptor and target cell somas was considered attraction. If there was no contact or no development of varicosities then process number and length toward and away from the target cell and changes in the distance between cells were the parameters used. Contacting processes were not included in this measure to avoid redundancy and because their growth potential was naturally limited by the contact. A minimum of two of the four types of measurements had to demonstrate differences to consider that attraction or inhibition had occurred. If quantification of growth did not reveal clear attraction or inhibition, the photoreceptor response to the target neuron was considered undetermined.

### Analysis of photoreceptor orientation

After placement, micromanipulated photoreceptors were at different orientations with respect to the target cell. Cell polarity for photoreceptors was determined by the position of the nucleus and the ellipsoid, the accumulation of mitochondria in the inner segment. Cell orientations were subdivided into three groups: 0 degrees (photoreceptor faced the target with its nuclear pole; the nuclear pole comprised the surface of the cell within 45 degrees either side of the center of the nucleus); 180 degrees (photoreceptor faced the target with its ellipsoid pole, defined as the surface of the cell 45 degrees either side of the center of the ellipsoid); and 90 degrees (cell was sideways). Changes in cell polarity were determined for each cell pair by comparing the orientation at day 1 with that at day 7.

### Statistical analysis

Graphs were created using SigmaPlot v.5.0 (Systat Software Inc., Chicago, IL). For percentage comparisons among groups exact binomial or χ^2^ analysis was employed using SAS version 8.0 (SAS Institute Inc., Cary, NC) software. Statistical comparisons of continuous measures between two groups used the Student's *t*-test if normality and equal variance tests were not rejected. Otherwise, the nonparametric Mann–Whitney rank sum test was used with SigmaStat v.2.0 (Systat Software Inc.). The data were expressed as mean ± standard error of the mean.

**Figure 5:**
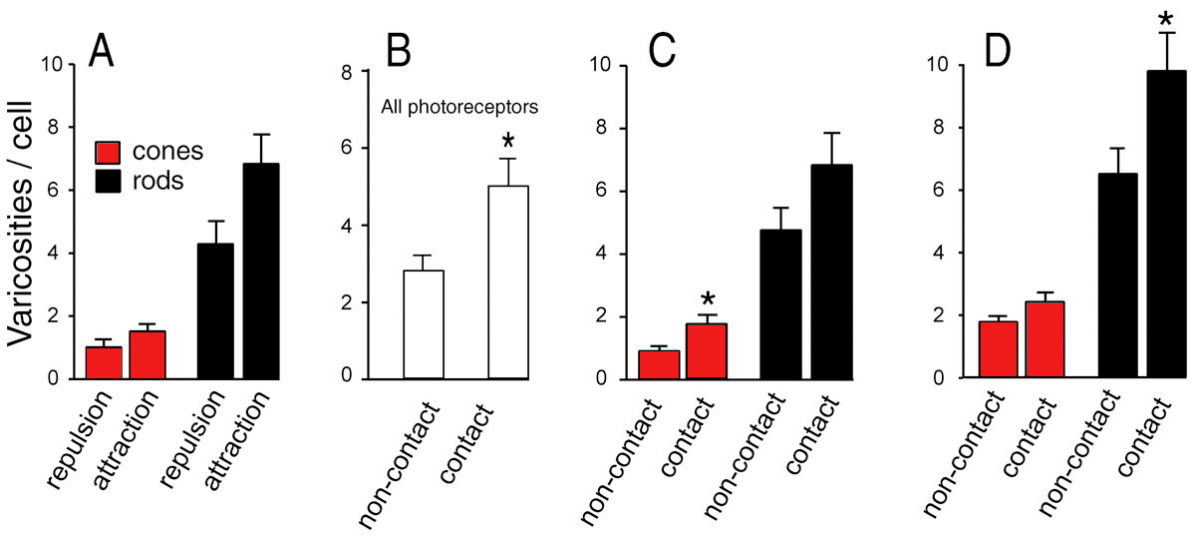
Varicosity development changes depending on cell pair interactions. **A:** For cones (n=105) and rods (n=133), the total number of varicosities did not depend on whether there was attraction or repulsion to the paired cell. The trend toward more varicosities in cells attracted to their target was not significant. **B:** However, the number of varicosities formed by photoreceptors was increased if contact with the target cell was present in one-week cultures (asterisk denotes p<0.05, tested with the Mann–Whitney test). **C:** Looking at the cone and rod cells separately, the average number of varicosities made by cone cells was significantly increased if contact was made with the target cell (asterisk denotes p<0.05, Mann–Whitney test; the n’s are the same as above). **D:** For rod cells, but not cones, the number of varicosities was significantly increased with contact when only those cells that made varicosities were analyzed (asterisk denotes p<0.05, Mann–Whitney test). Sixty-two of 105 cone cells had varicosities; 95 of 133 rod cells had varicosities.

## Results

### Interaction between photoreceptors and second- or third-order neurons

To examine the effect of cell type on photoreceptor targeting, we created pairs of rod-bipolar (second-order), rod-multipolar (third-order), cone-bipolar, and cone-multipolar cells. Optical tweezing was done within hours after retinal dissociation and cell plating and thus before any modification in cell shape. Although the neurons sustained some loss of cell processes during isolation, cell types remained distinct. Only cells positively identified were used to create cell pairs (see Methods). Cell identification was augmented by immunocytochemistry: anti-rod opsin immunolabeling distinguished rod from cone cells; anti-Goα antibody labeled most ON bipolar cells.

In culture, photoreceptors initially create actin-filled filopodia, which emanate from all points on the cell’s circumference [21]. Lamellipodia appear as well, frequently formed from existing synaptic pedicles [22]. Actin- and tubulin-filled neuritic processes develop from filopodia or regions of lamellipodia. Neurites subsequently develop synaptic-filled varicosities at their tips or along their length; varicosity-bearing processes can grow from any point along the cell soma [15,21]. Although the processes extend and retract, their growth is not controlled by typical growth cones, as would appear on projection neurons. The neurites extend a maximum of approximately 50 µm, which is two to three times cell soma diameter. Thus placing the photoreceptors within 2–10 µm of a target cell insured that the targets were well within their growth range. Cell pairs were followed for a week, a period of vigorous photoreceptor process growth but before functional synaptic development [8]. During this time photoreceptors grew neuritic processes, developed presynaptic varicosities, and in some cases formed cell contacts (Figure 1C), consistent with our previous studies [15]. Control pairs, created without micromanipulation and consisting of photoreceptors next to a second- or a third-order neuron, were identified in the same cultures used for tweezers micromanipulation.

Attraction or inhibition between cells can be initiated as well as maintained by secreted factors. To reduce the effects of extraneous soluble factors, we plated cultures at low density (range 16.3–343 cells/mm^2^, mean 90.6 cells/mm^2^) so that pairs were at least 100 µm away from other neurons. We discarded any pair in which a third neuron came in contact with one of the members. However, we made no attempt to limit diffusion from the target cells over the seven-day period and thereby enhance possible gradients of guidance factors [28,29]. Thus, with a prolonged period for diffusion of potential guidance factors, an all-or-nothing effect on the direction of photoreceptor growth was not anticipated.

The photoreceptors were analyzed by examining for cell contacts, and by quantifying changes in the number of presynaptic varicosities that formed toward or away from the partner, the number and total length of processes which grew toward or away from the potential postsynaptic partner, and the distance between cells over time. Although adult retinal neurons do not migrate in culture, in some cases of attraction, cells appeared to move together due to asymmetric expansion of the cell soma so that there was broad contact between cell bodies (Figure 2A). Based on these measures, a photoreceptor cell was classified as attracted to (Figures 1C, Figure 2A,B) or repulsed by (Figures 2C) its partner or undetermined. The undetermined category contained (1) photoreceptor cells that had neutral growth, i.e., equal amount of growth in all directions; (2) cells that had equal growth both toward and away from the target, i.e., cells that may have sensed multiple attractive or inhibitory molecules or may have been paired with a target cell which released weak or mixed signals; and (3) cells that responded poorly, i.e., cells that had little growth, making it difficult to assess growth patterns. We know from experience that about 1%–5% of the photoreceptors will not grow well in culture. We analyzed 203 pairs from 86 cultures derived from 55 animals.

Observation of the pairs throughout the seven days in vitro showed that retraction of neurites after process outgrowth was rare, suggesting that intercellular effects were relatively stable over time. Cone cells grew an average of 6.4±0.3 processes per cell and formed on average 1.4±0.1 presynaptic varicosities. From analysis of growth patterns, cone cells were found to be attracted to and repulsed by both bipolar and multipolar cells but in distinctly different proportions (Figure 3A). In cone-bipolar cell pairs (n=55), 53% showed attraction and 27% showed repulsion with the remaining pairs classed as undetermined; cones, therefore, showed an overall attraction to appropriate second-order targets. For pairs with third-order cells (n=43), 38% showed attraction and 56% showed repulsion, with 11% undetermined, suggesting a repulsion of cone cells to inappropriate multipolar targets. Rod cells grew an average of 35.3±1.5 processes and formed 5.8±0.6 varicosities per cell. For rod cells (Figure 3A), 52% were attracted and 28% were repulsed by their bipolar partner (n=74); like cone cells, rod cells showed an attraction to normal second-order targets. However, 65% of rod cells were attracted but only 10% were repulsed by multipolar partners (n=40), indicating an additional attraction to novel third-order cells. Of the pairs between rod and multipolar neurons showing attraction, 25% had formed broad cell-to-cell contact compared to 13% of attracted cone-multipolar cell pairs. This broad cell-cell contact was not due to fortuitous close pairing of cells: there was no statistical difference at day of plating in the intercellular distance between cells that subsequently showed attraction compared to those that subsequently showed repulsion. Moreover, pairs with broad contact were observed equally often in rod-bipolar and cone-bipolar cells (28% and 29% of attracted cell pairs, respectively), indicating that cone cells had the ability to make broad cell contacts. Attraction to multipolar cells by rod cells appeared to be strong both on the basis of the number of pairs showing attraction and the number of attracted pairs showing broad cell-cell contact. With chi-square analysis, the attraction of rod cells to multipolar cells was significantly greater than rod cells to bipolar cells (p=0.027, Figure 3B). Thus, there was an obvious difference in the effect of multipolar cells on growth and varicosity formation of cone and rod cells. This comparison between photoreceptor cell types is particularly striking when looking at the net effects of partners (% attraction minus % repulsion; see Figure 3B).

**Figure 6:**
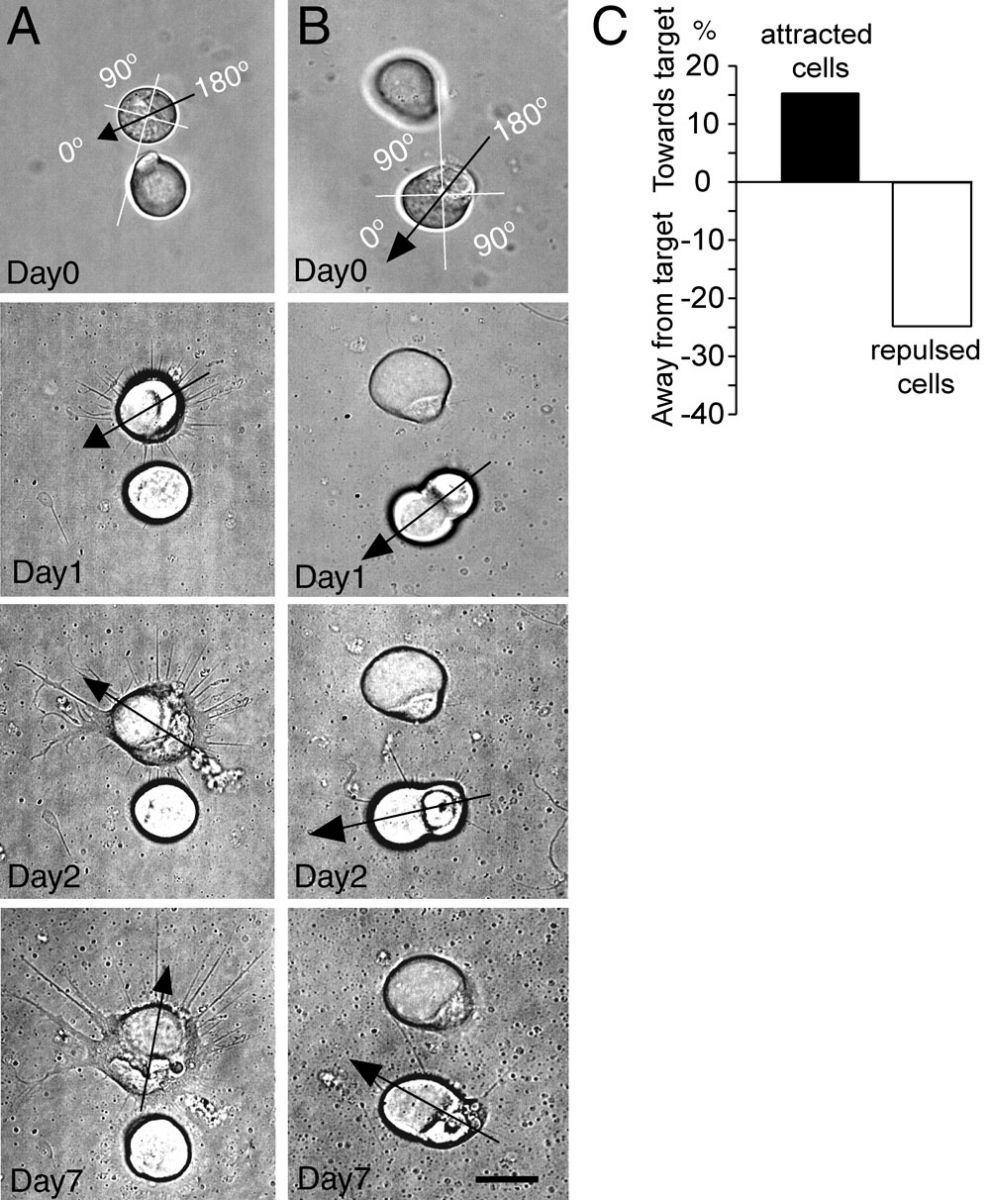
Photoreceptors change orientation to their target depending on whether there is attraction or repulsion. **A:** The axonal or nuclear pole of the cell was designated to be 0 degrees. Here a rod photoreceptor, repelled by its target cell, rotated over time so that the nuclear pole faced away from the target cell by seven days in culture. **B:** In this pair, a cone cell was plated at an orientation so that the nuclear pole was approximately 135 degrees away from the target cell. Over time, the cell rotated to bring the nuclear pole within about 45 degrees of the target cell. The cone was attracted to its target cell. Scale bar equals 20 µm. **C:** The difference between the percent which rotated toward and the percent which rotated away from their target cells was calculated for attracted (n=77) and repulsed pairs (n=51) to give the net percent of cells that rotated toward or away from their target cell. There was a significant association between attraction and repulsion and change in orientation of the cell (p=0.01) using χ^2^ analysis.

### Interactions between photoreceptors and bipolar cell subtypes

We speculated that although layer-specific markers associated with specific cell classes may determine targeting, cell subtypes may also influence the outcome of cell class pairings. To investigate the effects of cell subtype, we chose to examine photoreceptor-bipolar interactions because these second-order neurons are divided into two basic categories: ON and OFF cells. This division depends on the response to light: ON cells are active in the light; OFF cells are active in the dark. The functional differences are due in part to the differential presence of metabotropic and ionotropic glutamate receptors on ON and OFF cells respectively. In adult tiger salamander, ON and OFF cells are approximately equal in number [30]. Since, for both cone and rod cells, about half the bipolar cells were attractive targets, it is possible that either the ON or the OFF cells were the preferred target cell subtype within the bipolar cell class.

The same cultures as described were reexamined for bipolar subtype interaction by immunolabeling. In salamander retina, ON bipolar cells have either predominantly cone input, more equally mixed rod and cone input, or predominantly rod input [31] (Figure 4A). The presence of a unique receptor-associated G protein, Go, allowed us to positively distinguish most ON subtype cells. Staining for the alpha subunit of Go protein (Goα) is present in cone-dominated bipolars and mixed rod-cone bipolars, together comprising about 41% of all bipolar cells [26] (Figure 4A). The other 59% of bipolar cells, which are not immunoreactive for Goα, consists of the rod-dominated ON bipolars and all OFF cells. After fixation, cultures, which had been immunostained for rod opsin to distinguish rod from cone cells, were restained for Goα to distinguish ON and OFF bipolar cells (Figure 4B-G). There were 113 photoreceptor-bipolar pairs. For each category, attraction, repulsion, and undetermined, the number of Goα-positive and -negative cells were counted. Both Goα-positive and -negative bipolar cells were present in each category; however, Goα-positive cells were more attractive than repulsive (Figure 4H). For cones, 58% of Goα-positive cells were attractive, 29% were undetermined, and 13% were repulsive. For rod cells, 58% of Goα-positive cells were attractive, 23% were undetermined, and 19% were repulsive. χ^2^ analysis confirmed that cone and rod cells were more attracted than repulsed by Goα-positive cells (p<0.03 and p<0.02 respectively). In contrast, Goα-negative cells were approximately equally attractive and repulsive for both cone and rod cells (for cone cells, 44% versus 39%; for rod cells, 45% versus 35%). The data suggest a dependence upon bipolar cell subtype in neurite targeting, with the ON bipolar subtype providing a significantly attractive target for growth arising from both cone and rod cells. Thus, cell subtypes were not equally involved in targeting.

The number of Goα-positive cells present in the cone-bipolar cell pairs was greater than in the rod-bipolar cell pairs (Figure 4I). To ensure that the numbers of Goα-positive versus Goα-negative bipolar cells used as target cells did not skew the results, we examined the pool of bipolars presented to cone and rod cells. Based on staining in the intact retina, the ratio of Goα-positive:negative cells should be approximately 41%:59%. Unexpectedly, the pool of bipolar cells paired with cone cells contained 31 Goα-positive and 18 Goα-negative cells (Figure 4I), indicating that the Goα-positive cells were significantly more than 41% of the total pool (p<0.05). This is in contrast to the pool of cells paired with rod cells. For pairs between rod and bipolar cells there were 24 Goα-positive and 40 Goα-negative cells–close to the 41%:59% of Goα-positive:negative cells present in the intact retina. It is possible that cone cells caused Goα-negative cells to die; however, there were not adequate numbers of dying bipolar cells in all the created cone-bipolar cell pairs to account for the disproportionately large number of Goα-positive cells. Alternatively, cone cells may have stimulated or maintained an upregulation of Goα in bipolar cells.

For rod cells, even though less than 40% of the bipolar cell targets were Goα-positive, bipolar cells expressing Goα were more attractive than repulsive. The attraction of rod cells to Goα-positive cells is surprising when one considers the connectivity in the outer plexiform layer of the salamander retina. In salamander retina, all cone cells but only about 30% of rod cells (range 25%–35%) contact Goα-containing cells [26]. This circuitry would suggest that Goα-containing bipolars would have limited attraction for rod cells. Instead, rod cells in vitro were attracted to, instead of repulsed by, Goα -containing cells by almost 5:1 (14 attracted: 3 repulsed Goα-positive cells; Figure 4I). Thus, significantly more rod cells than expected (p=0.001) were attracted to Goα-containing cells. Further, it suggests that some rods were contacting novel cell subtypes.

### Effects of cell target on photoreceptor growth

The preferences demonstrated in culture may be influenced by general stimulation or inhibition of photoreceptor growth by potential postsynaptic partners. This would result in greater growth from photoreceptor cells attracted to their partner and possibly less growth with inhibition, which might make it more difficult to detect repulsion. We compared, therefore, the number of varicosities and the number and total length of processes produced by attracted versus repulsed photoreceptors. Surprisingly, repulsive cells did not reduce total photoreceptor growth. Instead, the average number of varicosities (Figure 5A), neurites, and total length of neuritic growth (data not shown) did not differ regardless of whether the cells were attracted to or repulsed by a partner cell. Additionally, qualitative assessment of the amount and direction of growth by target cells suggested that growth by target cells did not determine photoreceptor targeting: abundant growth by target cells toward the photoreceptor could still result in repulsion of photoreceptor growth, whereas no growth by a target cell could result in attraction (compare Figures 2B and 2C).

Although there were no statistical differences in the total amount of growth in attracted and repulsed pairs, there was a trend toward more neuritic development in attracted pairs. Previous work had shown an increase in the number of varicosities after cell contact in two-week-old cultures [15]. Thus we examined the effect of contact on photoreceptor growth parameters. In our one-week-old cultures, we found a significant increase in varicosities present in photoreceptors that contacted target cells (p<0.05; Figure 5B). About two-thirds of all photoreceptors that made contacts produced varicosities. When we examined the photoreceptors separately, we observed a significant increase (91%) in varicosities per cone cell with cell contact (Figure 5C) and per rod cell by 54%, with contact, if we looked only at rod cells that made varicosities (Figure 5D). The data suggest that regeneration of synaptic interaction occurs in two steps: (1) potential partners secrete a factor guiding neuritic growth but not determining total amount of growth; and (2) if contact is established, axonal differentiation in the form of presynaptic development is stimulated.

**Figure 7:**
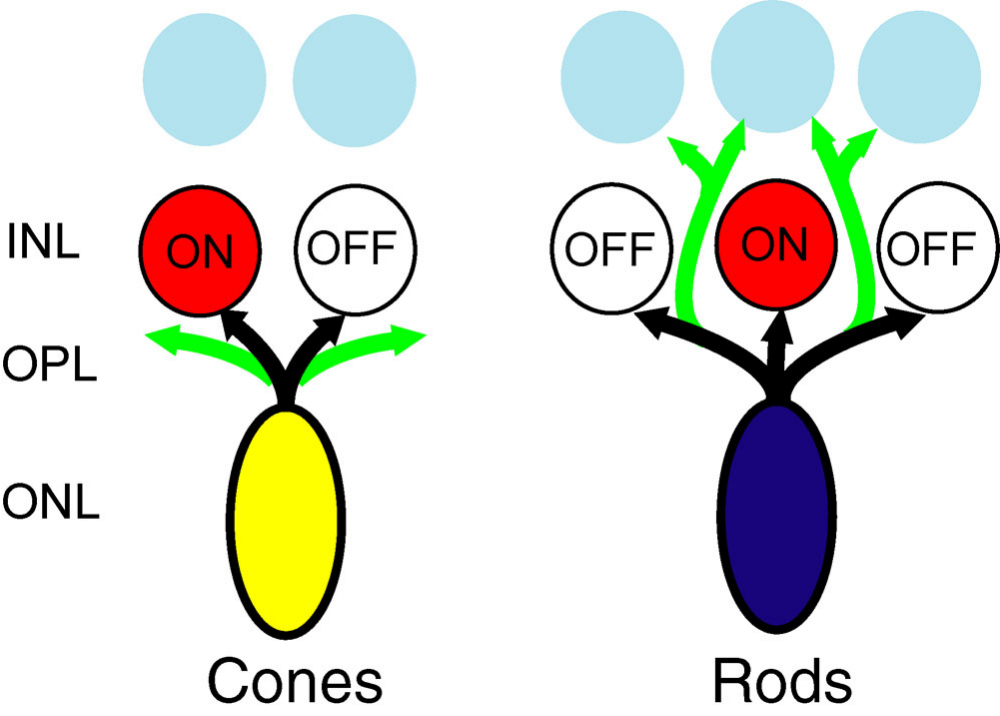
Attraction and repulsion of rod and cone neuritic growth to novel partners may contribute to the remodeling of retinal circuits in disease. In disease, as in vitro, rod cells are attracted to multipolar cells that lie in the inner nuclear layer (INL), whereas cone cells tend to form new growth near adjacent bipolar cells. Black arrows indicate normal axonal processes of photoreceptor cells in the retina; green arrows show new growth made in retinal disease. Other abbreviations used: OPL, outer plexiform layer; ONL, outer nuclear layer.

### Effects of cell target on photoreceptor polarity

Technical issues related to optical tweezing may also affect partner preferences. When cells are placed next to each other, the nuclear side of the photoreceptor from which the axon normally emerges does not always face the dendritic pole of the second- or third-order neuron. Because photoreceptors can grow processes from any point of the cell body [21], orientation was not expected to influence the direction of cell growth. However, to test for the effects of orientation, we analyzed cone and rod cells for polarity (location of the ellipsoid, an accumulation of mitochondria, with respect to the nucleus determines the axis of the cell). On days 1 and 7, where the nuclear pole faced, toward or away from the target cell, was assessed. No effect of initial polarity on final attraction or repulsion was seen. For example, less than a third of cones paired with a bipolar partner were correctly oriented one day after plating, whereas more than 40% of cone cells paired with multipolar cells was correctly oriented. If polarity determined preference, then cone cells should have preferred multipolar over bipolar cells; the opposite was in fact observed. By day 7, however, the polarity of some cell pairs unexpectedly had changed (Figure 6A and B). Changes were usually gradual, occurring over several days in culture. For photoreceptors whose polarity changed, there was a significant association between attraction and repulsion and change toward or away from the target respectively (p=0.01; Figure 6C).

Finally, pairs that were formed randomly in the culture dish, without the use of optical tweezers, were examined as internal controls for cell targeting (n=23). As previously reported [16], the use of optical tweezers did not change the morphology or amount of process outgrowth. The proportion of attracted and repulsed cell pairs based on cell type was not significantly different than for tweezers-manipulated pairs (p>0.05). The majority of rod cells, for instance, were attracted to multipolar cells.

## Discussion

Optical tweezers were used to pair identified cells under sterile culture conditions and test for regenerative interactions. In contrast to our previous study using randomly plated cells [15], with the tweezers we were able to follow cone and rod cells separately and create adequate numbers of pairs with bipolar and multipolar cells. Examination of pairs that grew for seven days demonstrated that cone and rod cells have different target preferences. Cone cells preferred to grow toward their normal partners, bipolar cells, when forming new neuritic sprouts; among bipolar cells, they were more attracted to ON than to OFF cells. In contrast, rod cells sought novel interactions. Their preferred partner was a third-order neuron, a novel target. Within the bipolar class, rod cells sought out Goα-positive bipolars, a bipolar subtype that normally interacts with only a fraction of the rod cells in vivo and, therefore, would also be a novel target to most rod cells. Thus, cell types considered to be closely related morphologically and functionally, demonstrated different abilities to target appropriately in culture.

This study also demonstrated the feasibility of using optical tweezers to examine neuronal growth. In our cultures nonmanipulated cell pairs had similar target preferences to pairs made by micromanipulation. Further, initial cell orientation, after placement of a cell by the tweezers, did not determine target preference. Thus, tweezers manipulation itself did not appear to influence cell-cell interactions. These techniques, therefore, should be applicable to any type of neuron and allow formation of groups of various sizes and composition.

The formation of neural circuits proceeds along a series of steps, which include axonal targeting to appropriate areas, tissue layers and cells, development of synaptic specializations, and activity-dependent refinement of synaptic connectivity. The present experiments examined a period in culture of robust growth preceding synapse formation and thus focused on the first phase of circuit reformation. It is possible that as synapses form between photoreceptors and their targets, preferences change, perhaps reducing the number of novel contacts between rod and multipolar cells. However, in two-week cultures when numerous presynaptic varicosities contact target cells and functional synapses are thought to be present [8], photoreceptors also preferred novel target cells [15]. Although this previous study did not distinguish cone from rod cells, it indicates that novel interactions are not lost as synapses form. Our results are reminiscent of observations from adult invertebrate cultures where some identified neurons are appropriately selective but others are promiscuous during synaptogenesis [32-34]. For rod cells, however, the promiscuity is not simply a demonstration of polyspecificity in which there is contact with both normal and novel targets, such as that seen in regenerating retinal ganglion cells [35]. Rod cells demonstrated a preference for novel targets.

During development, targeting preferences are influenced by multiple soluble and membrane-bound molecules produced by multiple cell types including glial cells. In vertebrate retina, the Sidekick family of proteins has been shown to be essential for development of some of the sublayers in the inner plexiform or synaptic layer [36]. Molecules that may determine targeting in developing outer synaptic layers of the neural retina include Sidekick-2 [36], integrins [37], dystroglycan [38] and N-cadherin (see review by Mumm et al. [39]). Whether any of these molecules plays a role in targeting after injury is unknown. In our system, there are few glia that survive the serum-free culture cultures; in addition by using low-density cultures, we reduced the number of sources of possible guidance compounds. Thus, the preferences seen in our cultures were most likely not due to glial-derived factors but to factors from the paired target cell. Because preferential growth was seen early on, often before cell contact, this suggests that some of the factors were secreted. Moreover, because no difference was seen in the total amount of growth with attractive versus repulsive targets we suggest that mature retinal neurons produce factors that act as guidance, or neurotropic, molecules, distinct from neurotrophic factors. The observed increase in varicosities after contact, however, may depend on neurotrophic effects. A stimulatory effect of contact has also been reported in adult nerve cell cultures from Aplysia [40]; in Aplysia, enhanced varicosity development depends on the release of a neurotrophic-like factor from the target cell [41]. Finally, guidance factors influenced not only where on the plasma membrane neuritic growth was initiated and whether or not neurites developed presynaptic varicosities but the polarity of the photoreceptor cell as well. Change in polarity occurred over several days and therefore is probably downstream to asymmetric neuritic outgrowth. In the future, using optical tweezers to create groups of photoreceptors and higher order neurons in conjunction with antibodies to molecular markers of developing retinal layers, it will be possible to identify and localize these laminar-specific molecules as well as other candidates known to be involved in laminar specific connections [7]. 

Cone and rod cells exhibited some similar and some dissimilar behaviors in culture. Both cone and rod cells changed their polarity in response to attractive or repulsive targets and increased varicosity formation after contact. However, there was a disparate effect of multipolar target cells on the direction of cone and rod cell growth. Multipolar cells attracted rod but repulsed cone cells. Targeting differences between the closely related cone and rod photoreceptors may be the result of different, but yet to be identified, presynaptic or nonsynaptic receptors. However, in addition, there are several known molecular differences in cone and rod synaptic terminals. There are cone- and rod-specific molecular mechanisms for vesicle exo- and endocytosis [42,43]; neurotransmission in cone and rod cells is linked to different kinds of L-type calcium channels [44,45]; calcium homeostasis is handled differently in the two cell types [46]; and cones alone have c-GMP-gated calcium channels at their terminal [47]. Intracellular calcium levels are involved in growth cone guidance in embryonic CNS neurons (for a review, see [48]). Differences in calcium homeostasis therefore may change responses to guidance factors. Levels of cyclic nucleotides also influence growth cone guidance. For instance, increased cGMP turns a repulsive guidance cue into an attractive one for *Xenopus* spinal neurons [49]; increased cAMP can overcome repulsion in adult mammalian spinal cord regeneration [50], whereas reduced protein kinase A activity can prevent regeneration of normal retinotectal topographic projections in fish [51]. Examination of cone- and rod-dominant retinas has revealed that cone and rod cells contain different levels of cyclic nucleotides; rod cells have higher levels of intracellular cGMP [52]. We have shown that neuritic sprouting by adult salamander cone cells in vitro is stimulated by cGMP but that rod cell sprouting is inhibited by cGMP [53]. The differing intracellular levels of cGMP and opposing effects of cGMP on cone and rod cell growth suggest that there are intrinsically different cGMP signaling pathways in cone and rod cells, which may in turn affect targeting after injury. We also have preliminary information that cAMP stimulates rod, but not cone, cell growth [54]. Thus, in addition to possible differences in cell surface receptors and cell specific ways of controlling calcium, differential cGMP and cAMP signaling may be underlying factors in the mechanisms which determine the disparate targeting of cone and rod cells.

Change in protein expression, a property of the injury response, may also affect adult cell targeting. By examining the common amino acid transmitters, we observed that the relative abundance of the neurochemical classes of second- and third-order salamander retinal neurons in culture was stable and equivalent to their relative proportions in intact retina [55]. In contrast, the expression of Goα appeared to change since the total number of Goα-positive bipolar cells paired with cone cells increased in culture. Subsequent studies with salamander neurons have demonstrated an approximately 60% increase in the number of Goα-expressing cells after 24 h in culture [56]. In the rd1 mouse, a model of human RP, the metabotropic glutamate receptors that associate with Goα have been shown to decrease in rod bipolar cells [57]. In this mouse model, the gene for the rod β subunit of cGMP-phosphodiesterase is mutated and rod photoreceptors degenerate. Thus, changes in bipolar receptor expression are presumed to be a transynaptic effect of rod cell degeneration. In retinal degenerations where some cone cells remain, rod bipolar cells may upregulate expression of ionotropic receptors [58]. In retinal detachment, a mechanical injury that separates the photoreceptors from the supporting retinal pigmented epithelium, a decrease in the expression of cone-specific proteins such as cone opsin has been observed [59]. In contrast, rod cell-specific protein expression is maintained [60]. In isolated and cultured salamander photoreceptors, as well, cone opsin expression is reduced (D. Sherry, personal communication) but rod opsin expression is maintained [61]. There is evidence, therefore, of changes in gene and protein expression in both postsynaptic and presynaptic retinal cells after injury, in vivo and in vitro.

Finally, in rod cells, the localization of protein changes as the outer segments degenerate. This is exemplified by the relocalization of rod opsin. Opsin, produced in the inner segment of the rod cells and added to outer segment membrane at the connecting cilium, is normally present in vesicles in the inner segment, discs of the outer segment and in the plasma membrane of the outer segment. In injury and disease and in isolated rod cells in culture, opsin is also found along the plasma membrane of the inner segment, cell body, and synaptic terminal [62-66]. Mislocalization of rod membrane protein may contribute to the propensity for novel interactions by rod cells.

Although the controlled environment of cell culture mimics intact tissue imperfectly, there is a striking parallel between our in vitro findings and the in vivo growth of photoreceptors in degenerating retina. In early stages of degeneration, rod cells grow extensive processes with varicosities out to the inner retina, where the dendrites of the third-order neurons lie. Sprouting from rod cells toward the inner retina is a robust phenomenon, seen in many forms of the human hereditary retinal disease, RP, in human retinal injury, in aging, and in human retinas with detachment and reattachment (for a review, see [12]). This pathology has been replicated in a cat model of retinal detachment/reattachment [67] and in an amphibian model of the autosomal-dominant form of RP [68]. Studies in human retinas and animal models have demonstrated that the presence of the normal target cells, bipolar and horizontal cells, does not deter rod cell neurites from seeking out inner retinal neurons. In contrast, human cone cells do not form extensive outgrowths into the inner retina. Further, in mouse and porcine animal models of RP, cone cells appear to form new synaptic connections with rod bipolar cells, Goα-containing cells (in mammals, all rod bipolars express Goα), in the outer plexiform layer [69] whereas mouse rod cells contact cone bipolars [70]. Thus, the preferences seen for growth in a controlled in vitro environment mimic the pattern for new sprouts in vivo (Figure 7) and suggest that cell-intrinsic mechanisms contribute to targeting observed in vivo.

Each type of cell pairing produced both positive and negative as well as undetermined responses. We attribute this to several possible factors. With retinal dissociation, retinal neurons are injured to varying degrees, and this may make them insensitive or less sensitive to guidance factors. Position in the retina may play a role in targeting, and cells from different locations, for instance nasal versus temporal retina, may be less inclined to interact. Finally, there are many subtypes of each cell class that show highly specific patterns of connectivity. Although we analyzed the red rod subtype (green rods form only a small group of the rod photoreceptors and do not stain for red rod opsin) and primarily looked at large single cones (double cones and small single cones are also present in salamander retina), the bipolar and multipolar cell groups we used do contain many neurochemical subtypes. Indeed, our previous study had shown that, in vitro, photoreceptors preferred GABAergic amacrine cell targets [15]. In human RP, a large proportion of sprouting rod neurites was also associated with GABAergic processes [71]. In ferret retina, during development, there are also sprouts from rod cells, which grow into the developing inner retina [72]. In this animal, the sprouts depend on the presence of cholinergic amacrine cells [73]. Because acetylcholine frequently colocalizes with GABA in retinal amacrine cells (e.g., see [74]), it is possible that GABA plays a role in rod neuritic growth during development as well. GABAergic amacrine cells, about half the population of salamander amacrine cells [75,76], may be more attractive to rod photoreceptors than other subtypes. Analysis of Goα-positive versus Goα-negative bipolar cells demonstrated that the Goα-positive ON bipolar subtype is generally more attractive to cone and rod cells. The effects of cell subtype may be more significant than injury or position in determining targeting within a cell class. Exactly what molecular constituent(s) associated with GABAergic amacrine and Goα-positive bipolar subtypes produces attraction remains to be determined. Effects of cell subtype, positional origin of cell (temporal versus nasal), and interaction among larger groups of cells are all future avenues of research using the optical tweezers approach.

Mature projection neurons show varying degrees of regenerative ability [77-80]. Even nerve cells in the CNS of cold-blooded vertebrates, renowned for their ability to regenerate, are not able to regenerate equally well [81,82] and mistargeting occurs [5]. In contrast to these studies, we have examined sensory neurons that regenerate local circuits via short presynaptic neurites, distinct from projection neurons in which axonal growth is guided by a growth cone. Nevertheless, our results support the view that there are intrinsic differences in the ability of neurons to reach normal targets after injury. We suggest that for photoreceptors the differences depend in part on injury-induced changes in protein expression and localization and inherent cell-specific differences in intracellular signaling mechanisms.

## References

[1] Holt CE, Harris WA (1998). Target selection: invasion, mapping and cell choice.. Curr Opin Neurobiol.

[2] Thanos S, Thiel HJ (1990). Regenerative and proliferative capacity of adult human retinal cells in vitro.. Graefes Arch Clin Exp Ophthalmol.

[3] Baird DH, Baptista CA, Wang LC, Mason CA (1992). Specificity of a target cell-derived stop signal for afferent axonal growth.. J Neurobiol.

[4] Isacson O, Deacon TW (1996). Specific axon guidance factors persist in the adult brain as demonstrated by pig neuroblasts transplanted to the rat.. Neuroscience.

[5] Meyer RL, Kageyama GH (1999). Large-scale synaptic errors during map formation by regeneration optic axons in the goldfish.. J Comp Neurol.

[6] Beazley LD, Sheard PW, Tennant M, Starac D, Dunlop SA (1997). Optic nerve regenerates but does not restore topographic projections in the lizard Ctenophorus ornatus.. J Comp Neurol.

[7] Sanes JR, Yamagata M (1999). Formation of lamina-specific synaptic connections.. Curr Opin Neurobiol.

[8] MacLeish (1988). PR, Townes-Anderson E. Growth and synapse formation among major classes of adult salamander retinal neurons in vitro.. Neuron.

[9] Lasansky A (1973). Organization of the outer synaptic layer in the retina of the larval tiger salamander.. Philos Trans R Soc Lond B Biol Sci.

[10] Lasansky A (1980). Lateral contacts and interactions of horizontal cell dendrites in the retina of the larval tiger salamander.. J Physiol.

[11] Wong-Riley MT (1974). Synaptic orgnization of the inner plexiform layer in the retina of the tiger salamander.. J Neurocytol.

[12] Townes-Anderson E, Zhang N. Synaptic plasticity and structural remodeling of rod and cone cells. In: Pinaud R, Tremere L, De Weerd P, editors. Plasticity in the Visual System: From Genes to Circuits. New York: Springer; 2006. p. 13–31.

[13] Marc R, Jones B, Watt C. Retinal remodeling: Circuitry revisions triggered by photoreceptor degeneration. In: Pinaud R, Tremere L, De Weerd P, editors. Plasticity in the Visual System: From Genes to Circuits. New York: Springer; 2006. p. 33–54.

[14] Fei Y (2002). Cone neurite sprouting: an early onset abnormality of the cone photoreceptors in the retinal degeneration mouse.. Mol Vis.

[15] Sherry DM, St Jules RS, Townes-Anderson E (1996). Morphologic and neurochemical target selectivity of regenerating adult photoreceptors in vitro.. J Comp Neurol.

[16] Townes-Anderson E, St Jules RS (1998). Sherry DM, Lichtenberger J, Hassanain M. Micromanipulation of retinal neurons by optical tweezers.. Mol Vis.

[17] Ashkin A (1991). The study of cells by optical trapping and manipulation of living cells using infrared laser beams.. ASGSB Bull.

[18] Ashkin A, Dziedzic JM, Bjorkholm JE, Chu S (1986). Observation of a Single-Beam Gradient Force Optical Trap for Dielectric Particles.. Opt Lett.

[19] Folkman J, Moscona A (1978). Role of cell shape in growth control.. Nature.

[20] MacLeish PR, Barnstable CJ, Townes-Anderson E (1983). Use of a monoclonal antibody as a substrate for mature neurons in vitro.. Proc Natl Acad Sci USA.

[21] Mandell JW, MacLeish PR, Townes-Anderson E (1993). Process outgrowth and synaptic varicosity formation by adult photoreceptors in vitro.. J Neurosci.

[22] Nachman-Clewner M, Townes-Anderson E (1996). Injury-induced remodelling and regeneration of the ribbon presynaptic terminal in vitro.. J Neurocytol.

[23] Hicks D, Molday RS (1986). Differential immunogold-dextran labeling of bovine and frog rod and cone cells using monoclonal antibodies against bovine rhodopsin.. Exp Eye Res.

[24] Sherry DM, Bui DD, Degrip WJ (1998). Identification and distribution of photoreceptor subtypes in the neotenic tiger salamander retina.. Vis Neurosci.

[25] Vardi N (1998). Alpha subunit of Go localizes in the dendritic tips of ON bipolar cells.. J Comp Neurol.

[26] Zhang J, Wu SM (2003). Goalpha labels ON bipolar cells in the tiger salamander retina.. J Comp Neurol.

[27] Nachman-Clewner M, St Jules R, Townes-Anderson E (1999). L-type calcium channels in the photoreceptor ribbon synapse: localization and role in plasticity.. J Comp Neurol.

[28] Lumsden AG, Davies AM (1983). Earliest sensory nerve fibres are guided to peripheral targets by attractants other than nerve growth factor.. Nature.

[29] Brown M, Keynes R, Lumsden A. The Developing Brain. New York: Oxford University Press Inc; 2001.

[30] Maple BR, Zhang J, Pang JJ, Gao F, Wu SM (2005). Characterization of displaced bipolar cells in the tiger salamander retina.. Vision Res.

[31] Wu SM, Gao F, Maple BR (2000). Functional architecture of synapses in the inner retina: segregation of visual signals by stratification of bipolar cell axon terminals.. J Neurosci.

[32] Haydon PG, Zoran MJ (1989). Formation and modulation of chemical connections: evoked acetylcholine release from growth cones and neurites of specific identified neurons.. Neuron.

[33] Camardo J, Proshansky E, Schacher S (1983). Identified Aplysia neurons form specific chemical synapses in culture.. J Neurosci.

[34] Schacher S, Rayport SG, Ambron RT (1985). Giant Aplysia neuron R2 reliably forms strong chemical connections in vitro.. J Neurosci.

[35] Scalia F (1987). Synapse formation in the olfactory cortex by regenerating optic axons: ultrastructural evidence for polyspecific chemoaffinity.. J Comp Neurol.

[36] Yamagata M, Weiner JA, Sanes JR (2002). Sidekicks: synaptic adhesion molecules that promote lamina-specific connectivity in the retina.. Cell.

[37] Li M, Sakaguchi DS (2004). Inhibition of integrin-mediated adhesion and signaling disrupts retinal development.. Dev Biol.

[38] Lunardi A, Cremisi F, Dente L (2006). Dystroglycan is required for proper retinal layering.. Dev Biol.

[39] Mumm JS, Godinho L, Morgan JL, Oakley DM, Schroeter EH, Wong RO (2005). Laminar circuit formation in the vertebrate retina.. Prog Brain Res.

[40] Glanzman DL, Kandel ER, Schacher S (1989). Identified target motor neuron regulates neurite outgrowth and synapse formation of aplysia sensory neurons in vitro.. Neuron.

[41] Hu JY, Goldman J, Wu F, Schacher S (2004). Target-dependent release of a presynaptic neuropeptide regulates the formation and maturation of specific synapses in Aplysia.. J Neurosci.

[42] Sherry DM, Heidelberger R (2005). Distribution of proteins associated with synaptic vesicle endocytosis in the mouse and goldfish retina.. J Comp Neurol.

[43] Wang MM, Janz R, Belizaire R, Frishman LJ, Sherry DM (2003). Differential distribution and developmental expression of synaptic vesicle protein 2 isoforms in the mouse retina.. J Comp Neurol.

[44] Taylor WR, Morgans C (1998). Localization and properties of voltage-gated calcium channels in cone photoreceptors of Tupaia belangeri.. Vis Neurosci.

[45] Morgans CW (2001). Localization of the alpha(1F) calcium channel subunit in the rat retina.. Invest Ophthalmol Vis Sci.

[46] Krizaj D, Lai FA, Copenhagen DR (2003). Ryanodine stores and calcium regulation in the inner segments of salamander rods and cones.. J Physiol.

[47] Rieke F, Schwartz EA (1994). A cGMP-gated current can control exocytosis at cone synapses.. Neuron.

[48] Henley J, Poo MM (2004). Guiding neuronal growth cones using Ca2+ signals.. Trends Cell Biol.

[49] Song H, Ming G, He Z, Lehmann M, McKerracher L, Tessier-Lavigne M, Poo M (1998). Conversion of neuronal growth cone responses from repulsion to attraction by cyclic nucleotides.. Science.

[50] Cai D, Qiu J, Cao Z, McAtee M, Bregman BS, Filbin MT (2001). Neuronal cyclic AMP controls the developmental loss in ability of axons to regenerate.. J Neurosci.

[51] Rodger J, Goto H, Cui Q, Chen PB, Harvey AR (2005). cAMP regulates axon outgrowth and guidance during optic nerve regeneration in goldfish.. Mol Cell Neurosci.

[52] Farber DB, Chase DG, Lolley RN (1980). Cyclic nucleotides in rod and cone-dominant retinas.. Neurochemistry International.

[53] Zhang N, Beuve A, Townes-Anderson E (2005). The nitric oxide-cGMP signaling pathway differentially regulates presynaptic structural plasticity in cone and rod cells.. J Neurosci.

[54] Townes-Anderson E, Chawla R, Zhang N. Differential roles of cAMP and cGMP in presynaptic plasticity of salamander cone and rod photoreceptors in vitro. ARVO Annual Meeting; 2003 May 4–9; Fort Lauderdale (FL).

[55] Sherry D, Townes-Anderson E. Neurotransmitter content of isolated adult retinal neurons during regenerative growth. ARVO Annual Meeting; 1995 May 14–19; Fort Lauderdale (FL)

[56] Clarke RJ, Ehrlich DJ, Elkabes S, Townes-Anderson E. Goα is upregulated in bipolar cells following the loss of their synaptic connections with photoreceptors ARVO Annual Meeting; 2007 May 6–10; Fort Lauderdale (FL).

[57] Strettoi E, Pignatelli V (2000). Modifications of retinal neurons in a mouse model of retinitis pigmentosa.. Proc Natl Acad Sci USA.

[58] Marc RE, Jones BW, Anderson JR, Kinard K, Marshak DW, Wilson JH, Wensel T, Lucas RJ (2007). Neural reprogramming in retinal degeneration.. Invest Ophthalmol Vis Sci.

[59] Rex TS, Fariss RN, Lewis GP, Linberg KA, Sokal I, Fisher SK (2002). A survey of molecular expression by photoreceptors after experimental retinal detachment.. Invest Ophthalmol Vis Sci.

[60] Rex TS, Lewis GP, Geller SF, Fisher SK (2002). Differential expression of cone opsin mRNA levels following experimental retinal detachment and reattachment.. Mol Vis.

[61] Zhang N, Townes-Anderson E (2002). Regulation of structural plasticity by different channel types in rod and cone photoreceptors.. J Neurosci.

[62] Lewis GP, Erickson PA, Anderson DH, Fisher SK (1991). Opsin distribution and protein incorporation in photoreceptors after experimental retinal detachment.. Exp Eye Res.

[63] Li ZY, Kljavin IJ, Milam AH (1995). Rod photoreceptor neurite sprouting in retinitis pigmentosa.. J Neurosci.

[64] Nir I, Haque R, Iuvone PM (2001). Regulation of cAMP by light and dopamine receptors is dysfunctional in photoreceptors of dystrophic retinal degeneration slow(rds) mice.. Exp Eye Res.

[65] Edward DP, Lim K, Sawaguchi S, Tso MO (1993). An immunohistochemical study of opsin in photoreceptor cells following light-induced retinal degeneration in the rat.. Graefes Arch Clin Exp Ophthalmol.

[66] Alfinito PD, Townes-Anderson E (2002). Activation of mislocalized opsin kills rod cells: a novel mechanism for rod cell death in retinal disease.. Proc Natl Acad Sci USA.

[67] Lewis GP, Charteris DG, Sethi CS, Fisher SK (2002). Animal models of retinal detachment and reattachment: identifying cellular events that may affect visual recovery.. Eye.

[68] Tam BM, Xie G, Oprian DD, Moritz OL (2006). Mislocalized rhodopsin does not require activation to cause retinal degeneration and neurite outgrowth in Xenopus laevis.. J Neurosci.

[69] Peng YW, Hao Y, Petters RM, Wong F (2000). Ectopic synaptogenesis in the mammalian retina caused by rod photoreceptor-specific mutations.. Nat Neurosci.

[70] Haverkamp S, Michalakis S, Claes E, Seeliger MW, Humphries P, Biel M, Feigenspan A (2006). Synaptic plasticity in CNGA3(−/−) mice: cone bipolar cells react on the missing cone input and form ectopic synapses with rods.. J Neurosci.

[71] Fariss RN, Li ZY, Milam AH (2000). Abnormalities in rod photoreceptors, amacrine cells, and horizontal cells in human retinas with retinitis pigmentosa.. Am J Ophthalmol.

[72] Johnson PT, Williams RR, Cusato K, Reese BE (1999). Rods and cones project to the inner plexiform layer during development.. J Comp Neurol.

[73] Johnson PT, Raven MA, Reese BE (2001). Disruption of transient photoreceptor targeting within the inner plexiform layer following early ablation of cholinergic amacrine cells in the ferret.. Vis Neurosci.

[74] Brecha N, Johnson D, Peichl L, Wassle H (1988). Cholinergic amacrine cells of the rabbit retina contain glutamate decarboxylase and gamma-aminobutyrate immunoreactivity.. Proc Natl Acad Sci USA.

[75] Yang CY, Yazulla S (1988). Localization of putative GABAergic neurons in the larval tiger salamander retina by immunocytochemical and autoradiographic methods.. J Comp Neurol.

[76] Deng P, Cuenca N, Doerr T, Pow DV, Miller R, Kolb H (2001). Localization of neurotransmitters and calcium binding proteins to neurons of salamander and mudpuppy retinas.. Vision Res.

[77] Richardson PM, Issa VM, Aguayo AJ (1984). Regeneration of long spinal axons in the rat.. J Neurocytol.

[78] Benfey M, Bunger UR, Vidal-Sanz M, Bray GM, Aguayo AJ (1985). Axonal regeneration from GABAergic neurons in the adult rat thalamus.. J Neurocytol.

[79] Xu XM, Zhang SX, Li H, Aebischer P, Bunge MB (1999). Regrowth of axons into the distal spinal cord through a Schwann-cell-seeded mini-channel implanted into hemisected adult rat spinal cord.. Eur J Neurosci.

[80] Borisoff JF, Pataky DM, McBride CB, Steeves JD (2000). Raphe-spinal neurons display an age-dependent differential capacity for neurite outgrowth compared to other brainstem-spinal populations.. Exp Neurol.

[81] Lyon MJ, Stelzner DJ (1987). Tests of the regenerative capacity of tectal efferent axons in the frog, Rana pipiens.. J Comp Neurol.

[82] Becker T, Bernhardt RR, Reinhard E, Wullimann MF, Tongiorgi E, Schachner M (1998). Readiness of zebrafish brain neurons to regenerate a spinal axon correlates with differential expression of specific cell recognition molecules.. J Neurosci.

